# Prostate Cancer awareness in the Lebanese population: a cross sectional national survey

**DOI:** 10.1186/s12889-021-11821-6

**Published:** 2021-09-25

**Authors:** Johnny Boustany, Maher Abdessater, Halim Akl, Anthony Kanbar, Joey El Khoury, Serge Assaf, Charbel Dabal, Charbel El Hachem, Antoine Kassis, Rodrigue Saad, Rami Halabi, Raghid El Khoury

**Affiliations:** 1 Urology department, Notre Dame des Secours University Hospital Center (CHUNDS), Byblos City, Lebanon; 2 School of medicine and medical sciences, Holy Spirit University of Kaslik (USEK), P.O. Box 446, Jounieh, Lebanon

**Keywords:** Prostate cancer, Awareness, Prevention, Early diagnosis, Lebanon

## Abstract

**Background:**

Prostate cancer (Pca) is the second most common cause of cancer among Lebanese men. Screening is an effective method to reduce prostate cancer mortality. This study assessed the knowledge, awareness, and screening practices among Lebanese population regarding Pca.

**Methods:**

A cross-sectional national study over all the Lebanese governorates on 1558 persons was undergone. Information on Pca knowledge and attitudes towards screening was obtained using a semi structured questionnaire. Bivariate and binary logistic regression were used to assess relations between Pca knowledge and socio-demographic characteristics.

**Results:**

The sample consisted of 1088 (69.8%) males and 470 (30.2%) females, with a mean age of 40 Y.O. Concerning early detection methods of Pca, its frequency, the ideal screening age, symptoms and curability of Pca, a significant difference (*p* < 0.05) was found when studying the following variables: the educational level (university level group having the highest percentage of correct answers), the individual monthly income (more correct answers were found with higher monthly income) and the age groups (most correct answers in the younger age groups).

**Conclusion:**

Our study points to the need of developing Pca prevention and education programs that should especially target older men, and people of low socioeconomic status and education in Lebanon. Such programs would increase awareness to Pca and screening, ultimately reducing the burden of advanced cancer through its early diagnosis.

## Introduction

Prostate cancer (Pca) is the second most common diagnosed malignancy in male after lung cancer [1]. According to the European association of urology (EAU), one over seven men worldwide will be diagnosed with Pca [2].

In addition, its incidence is increasing due to increased life expectancy and the evolution of the imaging techniques [3].

Although Pca is an age-related disease increasing after the sixth decade of life, its mortality rates are variable: high in African ethnicity, intermediate in the USA, and low in Asia [4].

A genetic predisposition is also identified; the probability of high risk Pca in men with relatives diagnosed with Pca is increased [5].

Screening and early detection of Pca is the main goal of the disease management, since it can reduce the mortality rate and the incidence of high risk Pca [2, 6].

The EAU guidelines recommends offering early PSA testing for men with elevated risk of Pca; these include men over 50 years of age, men over 45 years of age with a family history of Pca, and African-Americans over 45 years of age [2].

The American Urological Association recommends screening for 55 to 69 Y. O men. For men younger than age 55 years or older than 69 years, screening should be individualized [6].

In 2019, 174,650 new cases and a total of 31,620 deaths were assigned to the Pca in the United States of America [7].

In Lebanon, the total population is estimated to 6,100,000. In 2018, 17,294 new cases of cancer were recorded, and the number of cancer related deaths was 8976 [8].

Regarding Pca, 1503 new cases were diagnosed in 2018 (which is equivalent to 17,1% of all cancers in Lebanese men and to 8.7% of all cancers in both sexes), and 511 deaths were attributed to Pca [8].

Concerning Pca screening and early detection, it is recommended to offer prostate specific antigen (PSA) and digital rectal exam (DRE) for men with informed consent and requesting early diagnosis [2].

Many treatment modalities are approved depending on the Pca stage, but a higher curability rate was seen with early diagnosis of this malignancy [5]. This is why, and considering the importance of awareness on Pca, we designed this national survey to assess the knowledge of the Lebanese population regarding Pca, its risk factors, symptoms, and screening modalities.

Our aim was to investigate the awareness level regarding Pca and its screening, in the Lebanese society, and to identify factors affecting this awareness in our population.

## Material and methods

### Study design

This is a cross-sectional survey conducted between July 2020 and October 2020 on all the Lebanese territories, to assess the level of knowledge of the Lebanese population concerning Pca awareness.

Lebanese men and women of 18 years old and above were chosen randomly from all the Lebanese governorates. We excluded Lebanese citizens younger than 18 years old and men who were already diagnosed with prostate cancer.

### Assessment tool

A semi structured self-administered anonymous questionnaire was designed to achieve the goal of the study using prompt questions. It was divided into three parts; the first one consists of seven socio-demographic questions; the second part contains five questions assessing the knowledge level concerning Pca; and the last part has three questions for men above 50 years old to evaluate their own urological and medical history. The questionnaire is available upon request from the corresponding author.

In order to facilitate the comprehension of the medical terms, questions were written in two languages: English and Arabic. A pilot study of 35 men and 35 women has been initially conducted in order to examine the feasibility of our research endeavor. The format and the content were reviewed and approved by a group of experts.

Concerning the question about early Pca screening methods, we considered the answer to this question correct when the responders chose only “clinical bed test and blood tests”. The response was considered partially correct, when the answers were either clinical bed test or blood tests and false if any other combination was chosen.

Regarding the answers about the ideal age for Pca screening, based on the EAU 2019 guidelines, both answers “40 YO” and “50 Y.O”, were considered as correct [2].

Informed consent was obtained from all the participants to the survey which was approved by the Holy Spirit university of Kaslik ethical board, since it is in accordance with the declaration of Helsinki.

It is important to note that after the completion of the questionnaire, every participant received a leaflet including epidemiologic information about Pca, its risk factors, symptoms, ways of prevention and early detection.

### Sampling

Multi-stage sampling was used to select participants for this study: all Lebanese governorates were represented in proportion with the population density of each governorate. This staging makes the sample population representative of all the country.

The participants were randomly selected in mass screening events in all the country’s seven governorates. The questionnaires were available as a hardcopy and as an electronic copy using Microsoft Teams. All questionnaires were completed during the presence of at least one medical professional to clarify any misunderstanding.

### Statistical analysis

Descriptive statistics were performed to identify mean, standard deviation, and percentages. Data analysis was performed using both numerical and categorical scales. Categorical scales were used to divide responses into correct, partially correct and false.

We have used the Chi-square test as a statistical method to compare whether two qualitative variables are independent or not. ANOVA test was used to assess the impact of various demographic factors (age, education, and income) on Pca awareness. The relation between the two variables was considered statistically significant if *P*-value < 0.05 and the variables were considered independent if *P*-value > 0.05.

In addition, a Knowledge Index Score (KIS) was calculated based on correct answers to the five questions assessing knowledge concerning Pca. The calculated KIS was dichotomized based on equal percentiles. Correspondingly, a binary logistic regression was done with a backward LR method, using the KIS as a dependent variable.

Statistical analysis was performed using SPSS software on Windows (version 25.0, SPSS Inc., Chicago, Illinois).

## Results

### Socio-demographic data

In our study, 1558 successful responses were registered. The sample consisted of 1088 (69.8%) males and 470 (30.2%) females, equally spread over five brackets according to the responders’ age, with a mean of 40 Y.O.

Once we have determined the origin of every surveyed citizen, and in order to have a representative sample of the nation we weighed our results in accordance to the “Economic and Social Council of Lebanon”.

The majority of participants (56.8%) had high level education (University) and 26.9% had a monthly income in the lowest of the selected income categories (less than 450$). About the half (51%) of the participants were married. Demographic data is detailed in Table 1.

**Table 1:** Demographic data of the study population (*n* = 1558)

		Study population % (n)
Mean Age ± SD		40.4 ± 17.5
Gender	Male	69.8 (1088)
	Female	30.2 (470)
Marital status	Single	44.7 (697)
	Married	51.1 (796)
	Divorced	2.8 (44)
	Widow	1.4 (21)
Individual monthly income	< 450$	29.4 (458)
	450$to 900$	23 (358)
	900$ to 1350$	18 (280)
	1350$ to 1800$	17.7 (276)
	> 1800$	12 (187)
Education Level	Not educated	2.3 (36)
	School	10 (156)
	High school	30.6 (478)
	University	56.8 (886)

### Awareness and knowledge about Pca

When surveyed about the frequency of prostate cancer, 60.3% answered that Pca is a frequent disease, 9.7% considered it a rare disease and the answer of 30.1% was inconclusive.

Correct answers concerning knowledge about Pca are summarized in Table 2.

**Table 2:** Correct answers on Prostate cancer knowledge questions

Prostate Cancer Knowledge Questions	True answer (%)
Is the Pca a frequent disease?	939 (60.3%)
What are the early screening methods of Pca?	63 (4.1%)
What is the ideal screening age for Pca?	1094 (70.2%)
Is the Pca curable if early diagnosed?	1209 (77.6%)
Can Pca be diagnosed without any symptom?	587 (37.7%)

Concerning the question about early Pca, only 4.1% of the responses were correct, 53.8% were partially correct and 42.1% were false.

Regarding the answers about the ideal age for Pca screening, 32.7% (509 participants) chose 40 Y. O as the ideal age for Pca screening and 37.5% (585 participants) chose 50 Y. O as the ideal age to begin Pca screening.

When asked about whether prostate cancer can be diagnosed without any urological symptoms, 587 (37.7%) of the answers were positive, 434 (27.9%) answered that Pca cannot be diagnosed without any urological symptoms and 537 (34.5%) preferred not to answer this question.

Concerning early detection methods of Pca, its frequency, the ideal screening age, symptoms and curability of Pca, a significant difference (*p* < 0.05) was found when studying the following variables: the educational level (university level group having the highest percentage of correct answers), the individual monthly income (higher correct answers were found with higher monthly income) and the age groups (most correct answers in the younger age groups). Details of these results are found in Table 3.

**Table 3:** Stratification of Awareness of Prostate Cancer and Screening Modalities by Demographics

		Frequency of Pca		Curability of Pca		Pca screening methods		Ideal age for Pca screening		Urological symptoms with Pca	
		Frequent, % (n)	*p*	Yes, % (n)	*p*	Correct, % (n)	*p*	Correct, % (n)	*p*	Yes, % (n)	*p*
Age	[18; 22]	42 (134)	< 0.001	69.6 (222)	< 0.001	0.9 (3)	< 0.001	55.2 (176)	< 0.001	34.8 (111)	< 0.001
	[23;30]	67.4 (194)		77.8 (224)		4.2 (12)		68.8 (198)		41.7 (120)	
	[31;45]	66.7 (218)		79.2 (259)		6.7 (22)		73.4 (240)		37.8 (124)	
	[46;57]	67.3 (208)		81.9 (253)		3.9 (12)		81.9 (254)		43.7 (135)	
	> 58	58.4 (184)		79.7 (251)		4.4 (14)		71.7 (225)		30.9 (97)	
Gender	Male	57.9 (630)	0.01	75.7 (824)	0.01	4.1 (45)	0.74	70.3 (765)	0.88	37.5 (408)	0.93
	Female	65.7 (309)		82.1 (386)		3.8 (18)		69.9 (328)		38.1 (179)	
Marital status	Married	63.9 (509)	0.01	81.2 (646)	< 0.001	5.9 (47)	< 0.001	76.1 (605)	< 0.001	36.7 (292)	0.01
	Single	56.1 (391)		74.3 (518)		2.4 (17)		64.1 (447)		38.7 (270)	
	Widow	42.9 (9)		47.6 (10)		0.0 (0)		42.9 (9)		22.7 (5)	
	Divorced	68.2 (30)		81.8 (36)		0.0 (0)		72.7 (32)		45.5 (20)	
Educational level	Not Educated	41.7 (15)	< 0.001	47.2 (17)	< 0.001	0 (0)	< 0.001	56.8 (21)	< 0.001	24.3 (9)	< 0.001
	School	46.8 (73)		73.1 (114)		1.3 (2)		65.4 (102)		26.9 (42)	
	High school	51.5 (246)		70.9 (339)		1.5 (7)		63.2 (302)		34.9 (167)	
	University	68.2 (605)		83.3 (739)		6.1 (54)		75.4 (669)		41.6 (369)	
Monthly income	Less than 450$	50.8 (232)	< 0.001	70.5 (323)	< 0.001	2.2 (10)	< 0.001	61.6 (282)	< 0.001	31.4 (144)	< 0.001
	450$ to 900$	55.7 (199)		75.1 (269)		1.1 (4)		65.6 (235)		34.9 (125)	
	900$ to 1350$	64.3 (180)		78.3 (220)		5.4 (15)		71.1 (199)		36.4 (102)	
	1350$ to 1800$	71.7 (134)		87.2 (163)		8.0 (15)		82.4 (154)		45.7 (85)	
	More than 1800$	70.5 (194)		85.1 (235)		6.9 (19)		81.2 (224)		47.5 (131)	
Relative with Pca	No	80.7 (221)	< 0.001	86.5 (237)	< 0.001	4.7 (13)	0.229	77.0 (211)	< 0.001	52.9 (145)	< 0.001
	Yes	57.1 (662)		78.4 (909)		4.3 (50)		69.4 (804)		36.9 (428)	
	Don’t know	47.1 (57)		62.8 (76)		3.3 (4)		58.7 (71)		29.8 (36)	

We should mention that in the question concerning Pca symptoms, 35% of participants from the age group of 58 Y. O and older did not know that the Pca can be diagnosed without any urological symptoms.

Finally, we have found a significant difference (*p* < 0.01) in the gender of responders about Pca frequency and curability: females had a higher percentage of true answers concerning these two questions.

### Screening state of men above 50 Y.O

**Fig. 1:**
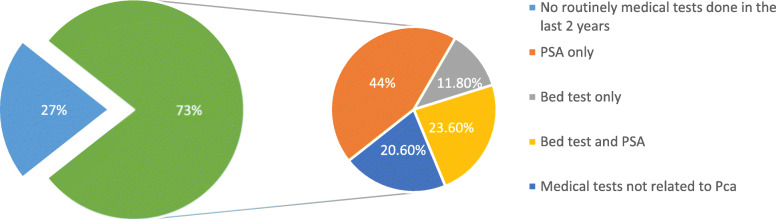
Screening tests undergone by men above 50 Y. O in the last 2 years

In the last part of our questionnaire addressed for men older than 50 years old, of the total 441 responders, 73% had undergone at least one routine medical exam during the last 2 years. Of the 73, 79.4% had medical exams for Pca screening. In details, 44% have only done the PSA blood test, 11.8% have undergone the clinical bed test exam only and 23.6% had both clinical and blood exam (Fig. 1).

The last question was addressed to men older than 50 years old who have not undergone any Pca screening test (20.6%): 26.4% of them did not know that it was necessary, 8.4% considered the annual tests expensive, 7.2% were afraid of the results.

#### Knowledge index score

The knowledge index score (KIS) was dichotomized based on equal percentiles. Of the total 1558 responders, 712 (45.7%) were considered having a low KIS and 846 (54.3%) were considered having a high KIS.

The results of the binary logistic regression using the KIS as a dependent variable and the socioeconomicals and family history of Pca as independent variables are presented in Table 4.

**Table 4:** Logistic regression using Backward LR method: knowledge Index Score is used as a dependent variable and socioeconomic data as independent variables

Independent Variable		O.R	95% CI	*p*-value
Age		1.011	1.001–1.02	0.025
Occupation (Medical is the reference)	Non-Medical Occupation	0.153	0.065–0.363	< 0.001
	Unemployed	0.113	0.046–0.277	< 0.001
Gender (Females is the reference)	Males	0.674	0.527–0.863	0.002
Education Level (Uneducated is the reference)	Primary	2.196	0.930–5.186	0.073
	Secondary	3.305	1.443–7.568	0.005
	University	4.936	2.166–11.249	< 0.001
Marital Status (Married is the reference)	Single	1.013	0.736–1.394	0.935
	Widowed	0.523	0.197–1.392	0.195
	Divorced	2.146	1.050–4.384	0.036
Individual Monthly Income (less than 450$ is the reference)	450$to 900$	1.023	0.747–1.402	0.887
	900$ to 1350$	1.103	0.778–1.564	0.583
	1350$ to 1800$	2.059	1.345–3.153	0.001
	> 1800$	1.458	0.991–2.143	0.055
Family history of Pca (having familial history is the reference)	No Family History	0.468	0.348–0.630	< 0.001
	Do not know about Family History	0.304	0.191–0.482	< 0.001

*OR* Odds Ratio. *C.I* Confidence Interval

Low KIS was associated with “non-medical” responders (OR = 0.153, *p* < 0.001) and unemployed ones (OR = 0.113, *p* < 0.001) compared to participants having a Medical occupation. Also, male gender had a lower KIS compared to females (OR = 0.674, *p* = 0.002). As well, having a family history of Pca resulted in higher scores in comparison for no Pca family history (OR = 0.468, p < 0.001). In contrast, an increase in KIS was remarked in participants with advanced age (OR = 1.011, *p* = 0.025). Also, being divorced had a higher KIS compared to married participants (OR = 2.146, *p* = 0.036). Lastly, having an individual monthly salary between 1350$ and 1800$ (OR = 2.059, *p* = 0.001) was associated to an increased KIS compared to participants having less than 450$ monthly salary.

## Discussion

This study is the first to assess the Lebanese population’s knowledge about Pca screening in order to know the level of awareness and the ability to undergo screening tests. Concerning early detection methods of Pca, its frequency, the ideal screening age, symptoms and curability of Pca, a significant difference (*p* < 0.05) was found when studying the following variables: the educational level (university level group having the highest percentage of correct answers), the individual monthly income (more correct answers were found with higher monthly income) and the age groups (most correct answers in the younger age groups).

To our knowledge, and after a rigorous literature review, there is no national surveys in Lebanon concerning Pca awareness, until these days. In addition, dedicated campaigns are not developed in our country. Therefore, we have been able to reveal gaps in awareness regarding Pca and its screening in Lebanon, with significant lack in knowledge about risk factors and symptoms of Pca. This alarming fact implies that men would not seek adequate medical attention, which would lead to the late diagnosis and treatment.

In this study, relatively young age (31 to 45 years), higher education, and high income were associated with higher level of awareness, indicating that lower socioeconomic groups should be especially targeted for Pca education. These results are in line with the study of Deibert et al. who have shown overall poor prostate cancer knowledge in low-income men [9]. Also, Campbell and McLain showed similar results: young adults were generally aware of the prostate specific antigen blood test, as well as risk factors for Pca [10].

Globally, only 4% of the Lebanese population knew the exact Pca screening tool, 70% knew the correct age to begin screening, and 38% knew that patients with Pca can be asymptomatic. This points to the fact that people do not consider Pca as a major health issue in their life and consequently Pca screening is undermined. When men begin to be aware of their increased risk for Pca, they will probably seek for medical advice concerning their prostate health and get screening by their medical doctor [11].

In addition, 20.6% of participants over the age of 50 years reported not being screened in the past 2 years. This can probably be linked to socioeconomic factors precluding these men from getting screened, specially that we observed a higher proportion of men over 50 years old getting screened among higher income individuals. Therefore, financial barriers to screening should be addressed by providing financially supported programs for Pca screening among individuals of lower socioeconomic status [12]. This would reduce the burden of Pca in Lebanon.

Moreover, a severe gap was identified in awareness of Pca risk in men with an affected relative: men at greatest risk were least likely to be aware. Consequently, there is a meaningful opportunity for education and screening that may benefit this population of high-risk patients who are unaware of their risk [13].

Also in multivariate analysis, similar results related to participants’ education were described by Deibert et al. However, low knowledge scores were associated with high age in opposition to our study results [9]. Findings concerning higher knowledge level among participants with higher monthly salary on multivariable analysis, were also described in the study of Woods-Burnham et al. [14].

Finally, our study has some limitations; this is a cross-sectional survey having a low-level of evidence. In addition, information bias may be encountered since the data collection was done by a face-to-face interview. Nevertheless, strengths of this study are also present; this is the first study to assess knowledge and to spread awareness concerning Pca in the Lebanese population. In addition, our sample size is large enough to be representative of all Lebanese governorates.

## Conclusion

In conclusion, this study points to the need of developing Pca prevention and control education programs in Lebanon. These programs should especially target older men, and people of lower socioeconomic status and education. Such actions would increase awareness to Pca and screening, ultimately reducing the burden of advanced cancer in Lebanon through earlier diagnosis and treatment.

It also highlights the unavoidable need of awareness campaigns, the importance to motivate general practitioners to put emphasis on Pca screening and consciousness, and the inevitable role of urologists to spread the knowledge about this preventable curable disease. On the other hand, further research should evaluate the potential opportunities for conversations between health care providers and patients in high-risk populations to help them make informed decisions about their screening and early interventions.

## Data Availability

The datasets used and/or analyzed during the current study are available from the corresponding author on reasonable request.
